# Supplementary material for: Dually modulated photonic crystals enabling high-power high-beam-quality two-dimensional beam scanning lasers
Source: Nat Commun. 2020 Jul 17;11:3487. doi: 10.1038/s41467-020-17092-w (PMC7367876; doi:10.1038/s41467-020-17092-w)
Supplement: Supplementary file 1 — Supplementary Information [file 41467_2020_17092_MOESM1_ESM.pdf]

Supplementary Information:
Dually modulated photonic crystals enabling high-power high-beam-quality two-dimensional beam scanning lasers

Ryoichi Sakata†, Kenji Ishizaki†, Menaka De Zoysa†, Shin Fukuhara, Takuya Inoue, Yoshinori Tanaka, Kintaro Iwata, Ranko Hatsuda, Masahiro Yoshida, John Gelleta, and Susumu Noda*

Department of Electronic Science and Engineering, Kyoto University, Kyoto 615-8510, Japan

† These authors contributed equally to this work

* Corresponding author: snoda@kuee.kyoto-u.ac.jp

CONTENTS

Supplementary Note 1. Difference between the concept proposed in this work and "diatomic" approach which was employed in the previous works

Supplementary Note 2. Difference between our device and a simple combination of independently fabricated 2D grating and photonic-crystal surface-emitting laser

Supplementary Note 3. Nano-antenna theory for dually modulated photonic crystal lasers

Supplementary Note 4. Parameters for calculations based on coupled-wave theory and the estimation of slope efficiency

Supplementary Note 5. Fabrication process of on-chip dually modulated photonic crystal lasers array

Supplementary Note 6. Operation details of real time 2D beam scanning

Supplementary Note 7. Device structure for beam scanning with a much larger number of resolvable points

Supplementary Note 8. Concept of a new, combined flash- and scanning-type LiDAR system

Supplementary Note 9. Estimation of emission angle change with respect to temperature

Supplementary References

Supplementary Note 1. Difference between the concept proposed in this work and "diatomic" approach which was employed in the previous works

Here, we explain the difference between the concept proposed in this work, "dually modulated photonic crystals" and the "diatomic" approach, which was employed in the previous works: The latter is basically based on pairs of photonic atoms, which are constructed, for example, by overlapping two different photonic structures, as in Supplementary Refs. 1 and 2. In the following, we will explain the difference between these two approaches by comparing the present work and those of Supplementary Refs. 1 and 2.

In Supplementary Ref. 1, the photonic crystal consists of overlapping square- and rectangular-lattice photonic crystals as shown in Supplementary Fig. 1(a), and forms a diatomic structure, where the interaction between a pair of photonic atoms in the combined crystal plays an important role: Due to the mutual interaction between a pair of photonic atoms, a new band-edge mode is formed, with which one-dimensional beam scanning is achieved. Next, in Supplementary Ref. 2, the developed photonic crystal is again a diatomic structure as shown in Supplementary Fig. 1(b), consisting of overlapping square-lattice photonic crystals separated by a shift in position. Due to the mutual interaction between the photonic atom pairs, the in-plane confinement strength of light is weakened (or, alternatively, the bandgap is reduced), through which large-area coherent resonance is achieved. Thus, these works can be considered to be based on a concept featuring the mutual interaction of a diatomic structure.

In contrast, the concept of the present paper does not concern diatomic structures. As proof of this, when we overlap one square-lattice photonic crystal with position modulation and another square-lattice photonic crystal with size modulation, a diatomic structure as shown in Supplementary Fig. 2(a) is produced. This structure is clearly different from the one developed in the present paper, which is reproduced in Supplementary Fig. 2(b) from Fig. 1a in the main text. The present structure is, so to speak, a "monoatomic" one, whose atoms have been individually modulated in position and size and exhibit no mutual interaction whatsoever with their neighbors. Namely, we engineered the individual photonic atoms of a monoatomic structure, which can be regarded as a nano-antenna array, to control radiation characteristics by manipulating interference of the electric fields of all photonic atoms of the

monoatomic structure in the far field, to realize ideal beam scanning lasers with high output power and high beam quality.

The difference between the diatomic structure (Supplementary Fig. 2(a)) and ours (Supplementary Fig. 2(b)) can be clarified even quantitatively. Supplementary Fig. 3 shows the radiation constants to free space and the total radiation constants in targeted steering angles $\theta = 17^{\mathrm{o}}$, 28°, 36°, and 54° for all possible lasing band-edge modes A-D of the diatomic structure and the present work's structure where azimuthal angle is set at $\phi = 0^{\mathrm{o}}/180^{\mathrm{o}}$. Here, note that the detailed derivation of radiation constants of individual band-edge modes is given in the successive main text as well as nano-antenna theory described in Supplementary Note 3, and thus we describe here the conclusion of the results to show the difference between our structure and the diatomic one. For our structure (Supplementary Fig. 3(e-h)), the radiation constants to free space (solid lines) are sufficiently large and almost equal to the total radiation constants (dashed lines), which include the additional portion of light that is radiated from the photonic crystal but trapped inside the device (such as the cladding layers and substrate). This means that the light of any band edge mode can be effectively radiated in all the targeted direction including even larger angles, which is important to realize fully 2D scanning of a high-power, high-quality beam. In contrast, for the diatomic structure (Supplementary Fig. 3(a-d)), the radiation constants to free space are smaller for all bands and, more importantly, far smaller than their corresponding total radiation constants, implying that much of the radiated light is trapped inside the cladding layers and substrate. These calculations demonstrate that our structure is able to steer a beam far more efficiently than a diatomic one, allowing us to conclude that these two structures differ not only in design, but also performance.

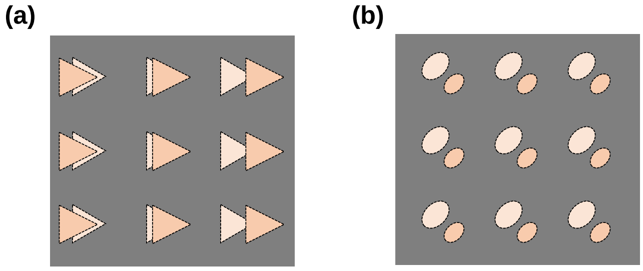

Supplementary Figure 1. (a) Diatomic structure of square lattice + rectangular lattice of Supplementary Ref. 1, (b) Diatomic structure of square lattice + (shifted) square lattice of Supplementary Ref. 2.

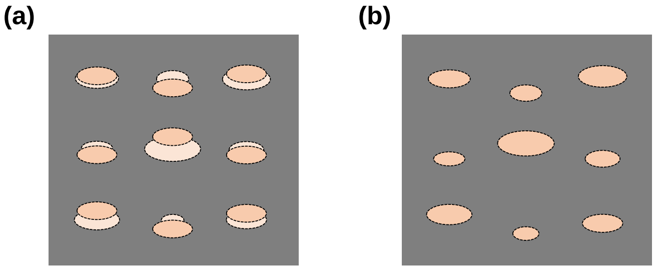

Supplementary Figure 2. (a) Diatomic structure of position-modulated square lattice + size-modulated square lattice, whose construction follows the same logic as that applied in the previous works of Supplementary Refs. 1 and 2, (b) Monoatomic structure (reproduced from Fig. 1a in the main text) of our current work.

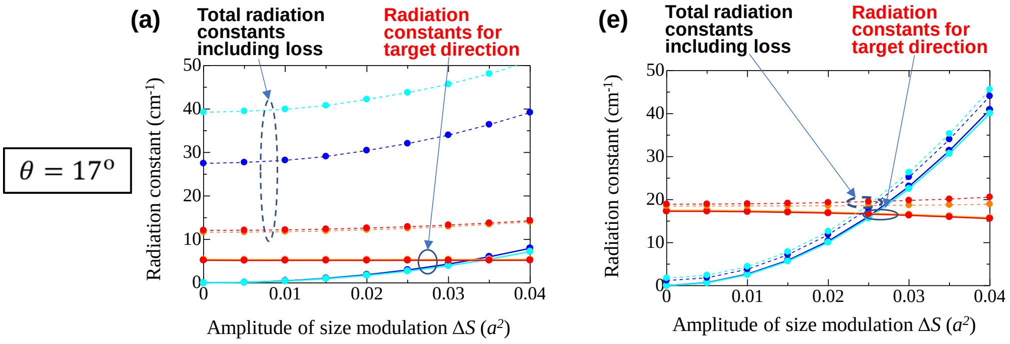

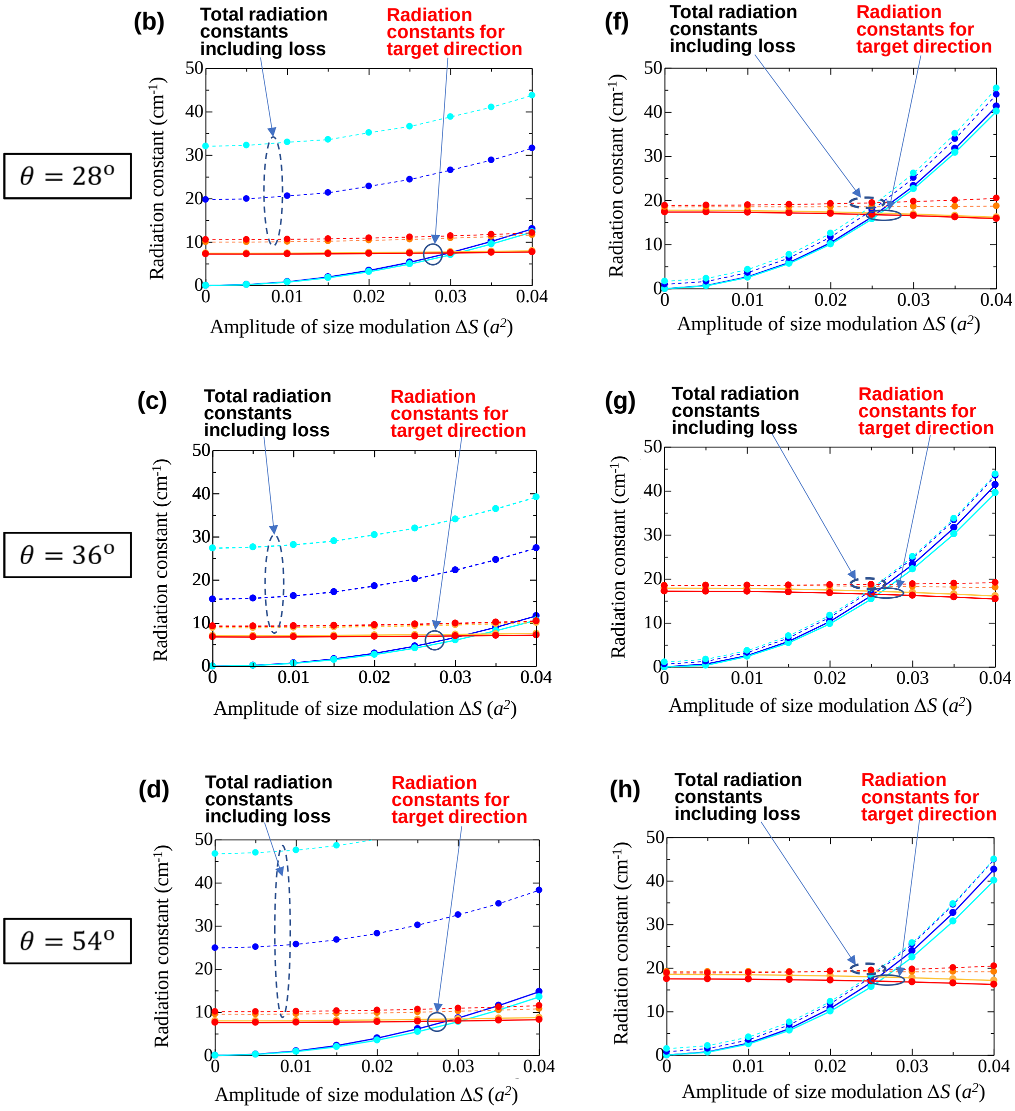

Supplementary Figure 3. Quantitative comparison of the radiation constants of (a-d) the diatomic structure and (e-h) the present work's structure. Steering (polar) angles are (a,e) $\theta = 17^{\rm o}$, (b,f) $28^{\rm o}$, (c,g) $36^{\rm o}$, and (d,h) $54^{\rm o}$, where azimuthal angle is set to $\phi = 0^{\rm o}/180^{\rm o}$. Radiation constants for individual band-edge modes (A-D) are shown as functions of size modulation ΔS with a fixed position modulation amplitude of $|\Delta \mathbf{d}| = 0.08a$. Colors of red, orange, light blue, and blue correspond to the band-edge modes A, B, C, and D, respectively. Solid lines indicate the radiation constant to free space, while dashed lines indicate the total radiation constant, which includes not only the radiation to freespace, but also that which is trapped inside the device (such as the cladding layers and substrate), where it is lost.

Supplementary Note 2. Difference between our device and a simple combination of independently fabricated 2D grating and photonic-crystal surface-emitting laser

In this section, we first analyze the simple combination of an independently fabricated 2D grating and a photonic-crystal surface-emitting laser (see Supplementary Fig. 4(a)), using the rigorous coupled wave analysis (RCWA) method. Here, the PCSEL is assumed to emit a Gaussian beam (or plane wave) with linear polarization. Supplementary Figs. 4(b) and (c) show examples of the calculated transmittance and reflectance, obtained using parameters chosen so that the desired beam directions by the $\pm 1^{st}$ order diffraction $|\theta|$ are 5° and 10°, respectively. Here, the $\pm m^{th}$-order diffraction angles $|\theta|$ by a grating with the period Λ can be expressed as follows: $|\theta| = \sin^{-1}[m(2\pi/\Lambda)/(2\pi/\lambda_0)]$, where λ_0 is the wavelength of the light. As shown in these figures, the beams are transmitted in not only the desired directions (=$\pm 1^{st}$-order diffraction), but also in the unwanted direction of 0° (0th-order diffraction), and even in unwanted directions corresponding to higher-order diffractions ($\pm 2^{nd}$-, $\pm 3^{rd}$-order beams, etc.). Reflected beams in the backward directions are also generated corresponding to diffractions of the 0th-, 1st-, 2nd-,3rd-order, and so on. We can change the intensity distribution among these orders by specially modifying the grating structure, but in general beams in the unwanted directions will remain. Supplementary Fig. 4(d) shows the calculated maximum transmittance by ±1st-order diffraction for a wide range of angles for an optimized rectangular grating structure. For the diffraction angles of $|\theta| < 30°$, where the higher-order diffractions exist, the maximum efficiency is less than 60% even for the optimized structure. The lower efficiency (<60%) hinders the realization of highly efficient beam scanning over the entire wide field of view, which is required in many practical applications. Furthermore, in LiDAR applications in particular, the emission of superfluous beams generated by 0th-, 2nd-, and higher-order diffractions will even induce noise in the reflected signal. The efficiency can be slightly improved at larger angles ($|\theta| > 30°$) where higher-order diffractions are prohibited, but even at these angles a relatively large portion of the incident power (~20%) is still lost as unwanted 0th and 1st-order reflection by the grating. Such unwanted reflection by the grating may even induce noise and instability in the lasing mode.

In contrast, our device is carefully designed so that such 0th-, 2nd-, and higher-order beams are not

emitted for any diffraction angle. Specifically, its lasing oscillation band edge is set at the M_1-point, which is outside the air-light cone, so that the zeroth-order beam is not emitted. In addition, the magnitude of the diffraction vector **k**, introduced by dually modulated photonic crystals, is set sufficiently large so that light can be emitted to free space from the M_1 point by first-order diffraction, but not by higher-order diffraction caused by 2**k,** 3**k,** and larger vectors. This is because the magnitudes of these vectors are too large to cause the light to be diffracted inside the air-light cone, and therefore high-order (e.g., 2nd-order, 3rd-order) beams cannot be emitted to free space.

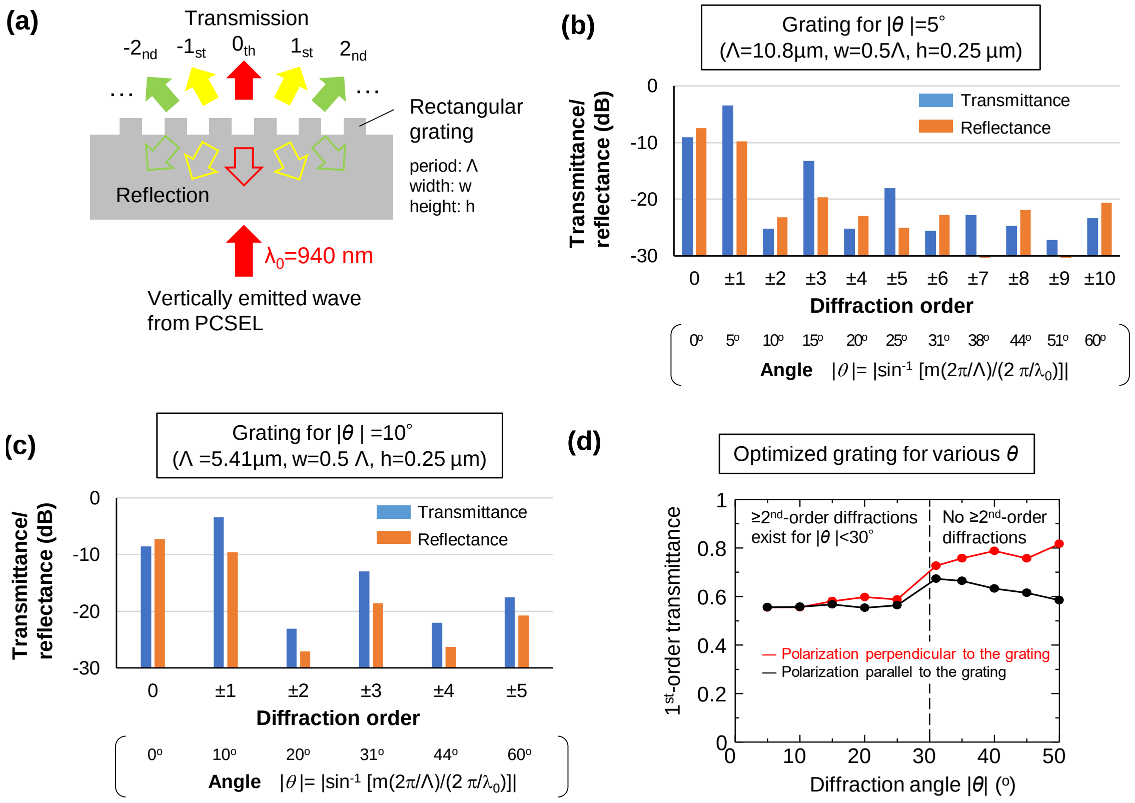

Supplementary Figure 4. (a) Schematics of beam diffraction with a rectangular grating on top of the vertically emitted PCSEL. (b), (c) Examples of calculated transmittance and reflectance for each beam diffracted by gratings designed to obtain desired beam directions of $|\theta| = 5^{o}$ and 10°, respectively, by $\pm 1^{st}$ order diffraction. (d) Calculated maximum transmittance in ±1st-order diffraction as a function of the diffraction angle $|\theta|$ when the polarization is perpendicular (red) and parallel (black) to the grooves of the grating.

Very recently, we realize that a meta-surface lens instead of a 2D grating is integrated on the substrate

of a VCSEL to focus and change the emission direction of the laser beam [Supplementary Ref. 3]. In this paper, although the generation of unwanted beams has been avoided by the use of a meta-surface lens, the obtained output power is limited to very small values (0.12mW), which is inherent to their strategy. In contrast, the output power of our device has reached watt-class (or even larger), which is more than four orders of magnitude higher than that of the above work for the same element size of 100μm. We may say that our work puts forth conceptual advancements compared with the above work, because our "dually modulated photonic crystals" possess all functions simultaneously, including 2D resonance, emission of light to any 2D direction, and keeping coherent oscillation over areas even as large as a 100μmϕ. Thus, the external-element-free, narrow-divergence, high-power, and high-beam-quality beam-scanning-operation becomes possible with our concept.

Supplementary Note 3. Nano-antenna theory for dually modulated photonic crystal lasers

Supplementary Note 3.1. Introduction

We formulate a nano-antenna theory, where we regard each air hole of a modulated photonic crystal as a nano-antenna and the modulated photonic crystal itself as an antenna array. In this theory, as the electric field of the resonant modes in photonic crystals, which is formed by the coupling of four fundamental waves, is partially radiated at the air holes, we assume that the air holes act as apertures.

Generally, the electric field radiated into the far field in a direction prescribed by its in-plane wave number $\mathbf{K} = \frac{2\pi}{\lambda}(\sin\theta\cos\phi, \sin\theta\sin\phi)$ is determined by the Fourier transform of its electric field distribution at the aperture $\mathbf{E}_{\text{aperture}}(\mathbf{r})$; to wit,

$$\mathbf{E}_{\text{far}}(\mathbf{K}) = \mathrm{C}\int\int \mathrm{d}x\mathrm{d}y\, \mathbf{E}_{\text{aperture}}(\mathbf{r})\exp(i\mathbf{K}\cdot\mathbf{r}) \quad (1)$$

Here, θ is the polar angle, ϕ is the azimuthal angle, and λ is the wavelength in free space. From Supplementary Eq. (1), it is clear that, in order to control $\mathbf{E}_{\text{far}}(\mathbf{K})$, it is necessary and sufficient to control $\mathbf{E}_{\text{aperture}}(\mathbf{r})$. In this formulation, we define $\mathbf{E}_{\text{aperture}}(\mathbf{r})$ as the portion of the electric-field distribution that overlaps each photonic-crystal air hole, then we perform a Fourier transform of this portion to obtain the electric field in the far field.

In Supplementary Note 3.2, we first derive the electric field distribution of the resonant mode on each of the band edges A, B, C and D at the M_1 point. In Supplementary Note 3.3, we derive the electric field distribution $\mathbf{E}_{\text{aperture}}(\mathbf{r})$ and its corresponding far field in a photonic crystal without modulation; it is shown that, in this case, the radiation of light to free space is forbidden for the resonant modes of all four band edges. Next, in Supplementary Note 3.4, we introduce position modulation to the air holes of the photonic crystal and derive its electric field in the far field, showing that radiation at oblique angles exists for the resonant modes of band edges A and B but not for those of band edges C and D. In Supplementary Note 3.5, we separately introduce size modulation to the air holes and show that, in contrast to position modulation, radiation at oblique angles exists for the resonant modes of band edges C and D but not for those of band edges A and B. In Supplementary Note 3.6, we introduce simultaneous position and size modulations to demonstrate that light can be radiated at oblique angles for the resonant

modes of all four band edges.

Supplementary Note 3.2. Electric field distribution at the M1 point in unmodulated photonic crystals

We first consider the resonant modes at the M1 point of an unmodulated photonic crystal. At the M1 point, the in-plane electric field distribution is formed by the four fundamental waves R_1, R_2, R_3 and R_4 (Shown in Supplementary Fig. 5). (For simplicity, we ignore here the *z* dependence of these basic waves and consider only their *x*- and *y*-components.)

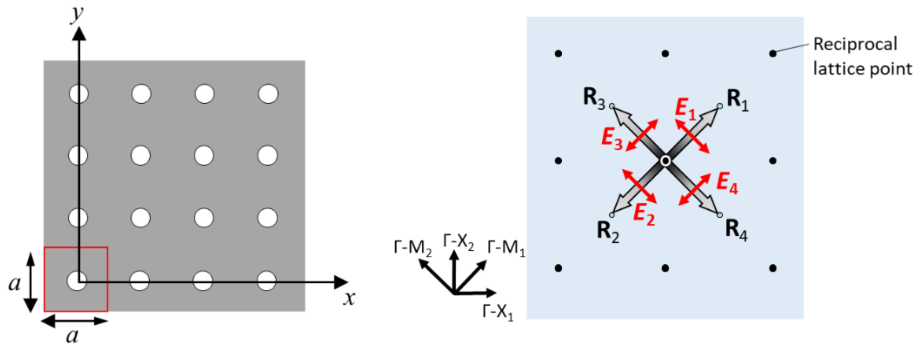

Supplementary Figure 5. Schematic of a square lattice photonic crystal and the electric-field vector of each fundamental wave.

The electric field distribution is formed by the four fundamental waves as follows:

$$\mathcal{E}(\mathbf{r}) = \sum_{i=1}^{4} \mathbf{E}_i \exp(i\mathbf{R}_i \cdot \mathbf{r}) \tag{2}$$

where $\mathbf{r}$ is a position vector and

$$\begin{gathered}
\mathbf{E}_1 = \left(-\frac{1}{\sqrt{2}}, \frac{1}{\sqrt{2}}\right) u_1,\ \mathbf{R}_1 = \left(\frac{1}{2}, \frac{1}{2}\right)\frac{2\pi}{a} \\
\mathbf{E}_2 = \left(\frac{1}{\sqrt{2}}, -\frac{1}{\sqrt{2}}\right) u_2,\ \mathbf{R}_2 = \left(-\frac{1}{2}, -\frac{1}{2}\right)\frac{2\pi}{a} \\
\mathbf{E}_3 = \left(-\frac{1}{\sqrt{2}}, -\frac{1}{\sqrt{2}}\right) u_3,\ \mathbf{R}_3 = \left(-\frac{1}{2}, \frac{1}{2}\right)\frac{2\pi}{a} \\
\mathbf{E}_4 = \left(\frac{1}{\sqrt{2}}, \frac{1}{\sqrt{2}}\right) u_4,\ \mathbf{R}_4 = \left(\frac{1}{2}, -\frac{1}{2}\right)\frac{2\pi}{a}
\end{gathered} \tag{3}$$

where a is a lattice constant.

Here, $(u_1, u_2\ u_3\ u_4) \equiv \mathbf{u}$ is the eigenvector of the coupled-wave equations and is determined by the structure of the photonic crystal. Since four fundamental waves exist, four eigenmodes also exist; we label these modes A, B, C and D, after the band edges on which they reside. Specifically, the eigenvectors exhibit the following relations:

$$\begin{aligned} \mathbf{u}_\mathrm{A} &\propto (+1, +1, +1, +1) \\ \mathbf{u}_\mathrm{B} &\propto (-1, -1, +1, +1) \\ \mathbf{u}_\mathrm{C} &\propto (+1, -1, +1, -1) \\ \mathbf{u}_\mathrm{D} &\propto (+1, -1, -1, +1) \end{aligned} \tag{4}$$

This vector $\mathbf{u}$ defines the relationship of position (or phase) between the air holes and the electric field distribution. In Supplementary Fig. 6, we illustrate the electric field distribution of each band edge around the photonic crystal air holes.

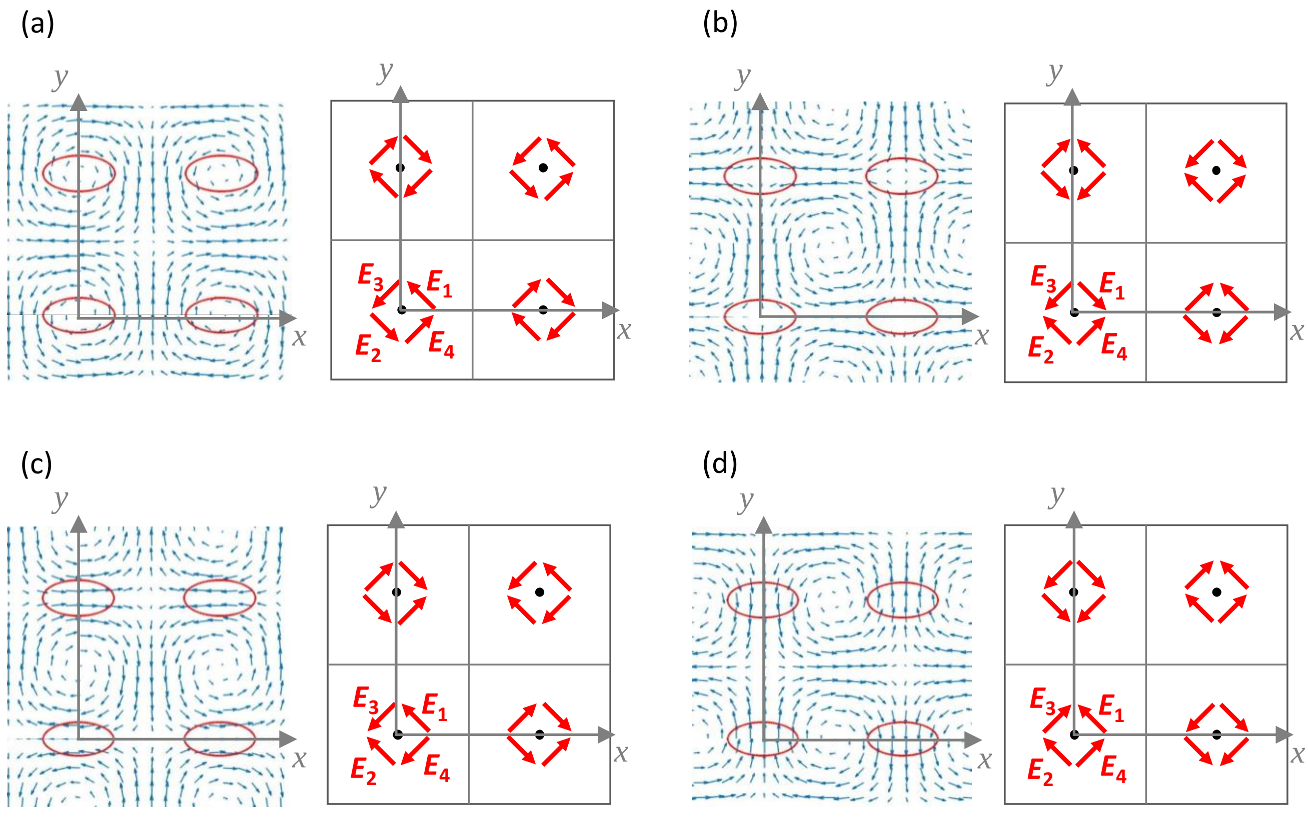

Supplementary Figure 6. Electric field distribution of band edges (a) A, (b) B, (c) C and (d) D.

As shown in this figure, an electric-field node lies at the center of each air hole for band edges A and B, and, conversely, an electric-field antinode lies at the center of each air hole for band edges C and D. This is also clear in the following expressions (i) and (ii) for $\mathcal{E}(\mathbf{r})$.

(i) Band edges A and B : $\mathbf{E}_1 = -\mathbf{E}_2, \mathbf{E}_3 = -\mathbf{E}_4$, so

$$\begin{aligned}\mathcal{E}(\mathbf{r}) &= \sum_{i=1}^{4} \mathbf{E}_i \exp(i\mathbf{R}_i \cdot \mathbf{r}) \\ &= \mathbf{E}_1\exp(i\mathbf{R}_1 \cdot \mathbf{r}) + \mathbf{E}_2\exp(i\mathbf{R}_2 \cdot \mathbf{r}) + \mathbf{E}_3\exp(i\mathbf{R}_3 \cdot \mathbf{r}) + \mathbf{E}_4\exp(i\mathbf{R}_4 \cdot \mathbf{r}) \\ &= \mathbf{E}_1\exp(i\mathbf{R}_1 \cdot \mathbf{r}) - \mathbf{E}_1\exp(-i\mathbf{R}_1 \cdot \mathbf{r}) + \mathbf{E}_3\exp(i\mathbf{R}_3 \cdot \mathbf{r}) - \mathbf{E}_3\exp(-i\mathbf{R}_3 \cdot \mathbf{r}) \\ &= 2i\mathbf{E}_1 \sin(\mathbf{R}_1 \cdot \mathbf{r}) + 2i\mathbf{E}_3 \sin(\mathbf{R}_3 \cdot \mathbf{r})\end{aligned} \quad (5)$$

The geometric center of each air hole is given by $\mathbf{r}^0_{m,n} = (ma, na)$, where m and n are integers. By using the relationship $\mathbf{R}_1 \cdot \mathbf{r}^0_{m,n} = \frac{2\pi}{a}\left(\frac{1}{2} \cdot ma + \frac{1}{2} \cdot na\right) = \pi(m+n)$, we see that

$$\begin{aligned}\mathcal{E}(\mathbf{r}^0_{m,n}) &= 2i\mathbf{E}_1 \sin(\mathbf{R}_1 \cdot \mathbf{r}^0_{m,n}) + 2i\mathbf{E}_3 \sin(\mathbf{R}_3 \cdot \mathbf{r}^0_{m,n}) \\ &= 2i\mathbf{E}_1 \sin(m\pi + n\pi) + 2i\mathbf{E}_3 \sin(m\pi + n\pi) \\ &= 0\end{aligned} \quad (6)$$

This expression states that an electric-field node exists at the center of each air hole, exactly as observed in Supplementary Figs. 6(a) and (b).

(ii) Band edges C and D : $\mathbf{E}_1 = \mathbf{E}_2, \mathbf{E}_3 = \mathbf{E}_4$, so

$$\begin{aligned}\mathcal{E}(\boldsymbol{r}) &= \sum_{j=1}^{4} \mathbf{E}_j \exp(i\mathbf{R}_j \cdot \mathbf{r}) \\ &= 2\mathbf{E}_1 \cos(\mathbf{R}_1 \cdot \mathbf{r}) + 2\mathbf{E}_3 \cos(\mathbf{R}_3 \cdot \mathbf{r})\end{aligned} \quad (7)$$

Substituting $\mathbf{r}^0_{m,n} = (ma, na)$ as before, we obtain the following equation.

$$\begin{aligned}\mathcal{E}(\mathbf{r}^0_{m,n}) &= 2\mathbf{E}_1 \cos(\mathbf{R}_1 \cdot \mathbf{r}^0_{m,n}) + 2\mathbf{E}_3 \cos(\mathbf{R}_3 \cdot \mathbf{r}^0_{m,n}) \\ &= 2\mathbf{E}_1 \cos(m\pi + n\pi) + 2\mathbf{E}_3 \cos(m\pi + n\pi) \\ &= 2(-1)^{\mathrm{m+n}}\mathbf{E}_1 + 2(-1)^{\mathrm{m+n}}\mathbf{E}_3\end{aligned} \quad (8)$$

This expression shows that an electric-field antinode exists at the center of each air hole, and additionally

that the electric fields at adjacent holes are of opposing polarity, exactly as observed in Supplementary Figs. 6(c), (d).

Supplementary Note 3.3. Far-field electric field properties of unmodulated photonic crystals

In Supplementary Note 3.2, we have considered an electric-field distribution formed by the propagation of basic waves in the plane of the photonic crystal. Now, in this section, we consider the out-of-plane scattering of these fundamental waves at each air hole. We define this scattered electric field distribution as $\mathbf{E}_{\text{aperture}}(\mathbf{r})$, from which we calculate the electric field in the far field, $\mathbf{E}_{\text{far}}(\mathbf{K})$.

Let us assume that the fundamental waves propagating in the photonic crystal are radiated only at the air holes, such that

$$\mathbf{E}_{\text{aperture}}(\mathbf{r}) = h(\mathbf{r})\boldsymbol{\mathcal{E}}(\mathbf{r}) \tag{9}$$

where

$$h(\mathbf{r}) = 1 \ \ (\text{for } \mathbf{r}\text{ : inside air hole})$$

$$h(\mathbf{r}) = 0 \ \ (\text{for } \mathbf{r}\text{ : outside air hole})$$

By substituting this relationship into Supplementary Eq. (1), we obtain

$$\begin{aligned}\mathbf{E}_{\text{far}}(\mathbf{K}) &= C\int\int \mathrm{d}x\mathrm{d}y\, h(\mathbf{r})\boldsymbol{\mathcal{E}}(\mathbf{r})\exp(i\mathbf{K}\cdot\mathbf{r}) \\ &= C\sum_{n}\sum_{m}\int\int_{\text{airhole}} \mathrm{d}x'\mathrm{d}y'\,\boldsymbol{\mathcal{E}}(\mathbf{r}'+\bar{\mathbf{r}}_{m,n})\exp(i\mathbf{K}\cdot\mathbf{r}')\exp(i\mathbf{K}\cdot\bar{\mathbf{r}}_{m,n})\end{aligned} \tag{10}$$

where $\bar{\mathbf{r}}_{m,n}$ is the position vector of the geometric center of each air hole and $\mathbf{r}' = \mathbf{r} - \bar{\mathbf{r}}_{m,n}$ represents the coordinate system whose origin lies at $\bar{\mathbf{r}}_{m,n}$. Next, we integrate over the air holes in Supplementary Eq. (10). As the phase change of $(\mathbf{K}\cdot\mathbf{r}')$ is small, we approximate $\exp(i\mathbf{K}\cdot\mathbf{r}')\sim 1$. $\mathbf{E}_{\text{far}}(\mathbf{K})$ then becomes

$$\mathbf{E}_{\text{far}}(\mathbf{K}) = \sum_{n}\sum_{m}\bar{S}_{m,n}\,\boldsymbol{\mathcal{E}}_{\text{ave}}(\bar{\mathbf{r}}_{m,n})\exp(i\mathbf{K}\cdot\bar{\mathbf{r}}_{m,n}) \tag{11}$$

$$\boldsymbol{\mathcal{E}}_{\text{ave}}(\bar{\mathbf{r}}_{m,n}) = \frac{1}{\bar{S}_{m,n}}\int\int_{\text{airhole}} \mathrm{d}x'\mathrm{d}y'\,\boldsymbol{\mathcal{E}}(\mathbf{r}'+\bar{\mathbf{r}}_{m,n}) \tag{12}$$

where $\bar{S}_{m,n}$ is the area of each air hole at $\bar{\mathbf{r}}_{m,n}$. Here, we defined $\boldsymbol{\mathcal{E}}_{\text{ave}}(\bar{\mathbf{r}}_{m,n})$ as the averaged electric

field inside the air hole at $\bar{\mathbf{r}}_{m,n}$. From Supplementary Eq. (11), we see that the radiation characteristics are determined by $\bar{S}_{m,n}$ and $\mathcal{E}_{\text{ave}}(\bar{\mathbf{r}}_{m,n})$.

Next, we show in Supplementary Fig. 7 the calculated value of $|\mathcal{E}_{\text{ave}}(\bar{\mathbf{r}}_{m,n})|$ when $\bar{\mathbf{r}}_{m,n} = \mathbf{r}^0_{m,n} = (ma, na)$, where m and n are integers, as a function of air-hole size $\bar{S}_{m,n} = S$. Here, in the limit of $S \to 0$, $\mathcal{E}_{\text{ave}}(\mathbf{r}^0_{m,n})$ is equal to $|\mathcal{E}(\mathbf{r}^0_{m,n})|$. From Supplementary Fig. 7, for band edges A and B, $|\mathcal{E}_{\text{ave}}(\mathbf{r}^0_{m,n})|$ is zero for all air-hole sizes because electric-field nodes reside at each air hole, as explained in Supplementary Note 3.2. Meanwhile, for band edges C and D, $|\mathcal{E}_{\text{ave}}(\mathbf{r}^0_{m,n})|$ gently decreases as S increases. Nevertheless, $|\mathcal{E}_{\text{ave}}(\mathbf{r}^0_{m,n})|$ decreases by only around 20% even when S increases from 0 to $0.2a^2$. Thus, for simplicity, we shall assume that $|\mathcal{E}_{\text{ave}}(\bar{\mathbf{r}}_{m,n})|$ is approximately equal to $|\mathcal{E}(\bar{\mathbf{r}}_{m,n})|$ when $S < 0.2a^2$:

$$\mathcal{E}_{\text{ave}}(\bar{\mathbf{r}}_{m,n}) \cong \mathcal{E}(\bar{\mathbf{r}}_{m,n}) \tag{13}$$

From Supplementary Eqs. (11) and (13),

$$\mathbf{E}_{\text{far}}(\mathbf{K}) \cong C \sum_n \sum_m \bar{S}_{m,n}\, \mathcal{E}(\bar{\mathbf{r}}_{m,n}) \exp(i\mathbf{K} \cdot \bar{\mathbf{r}}_{m,n}) \tag{14}$$

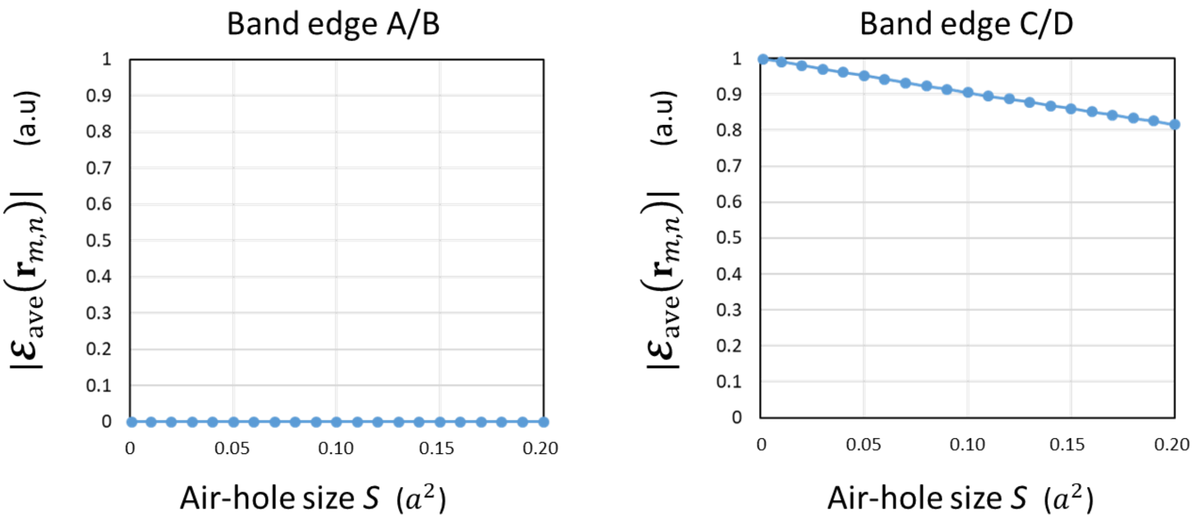

Supplementary Figure 7. Amplitude of the radiative electric field $|\mathcal{E}_{\text{ave}}(\boldsymbol{r}_{m,n})|$ from each air hole per unit area as a function of air-hole size S.

By substituting the electric field distributions in Supplementary Eqs. (5) and (7) into Supplementary Eq. (14), we obtain the following equations:

(i) Band edges A and B :

$$\mathbf{E}_{\text{far}}(\mathbf{K}) = 0 \quad (\text{Because } \mathcal{E}(\mathbf{r}^0_{m,n}) = 0) \tag{15}$$

(ii) Band edges C and D :

$$\begin{aligned}\mathbf{E}_{\text{far}}(\mathbf{K}) = C\sum_n\sum_m S_{m,n}\cdot\mathbf{E}_1\cdot\{\exp(i(\mathbf{K}+\mathbf{R}_1)\cdot\mathbf{r}_{m,n}) + \exp(i(\mathbf{K}-\mathbf{R}_1)\cdot\mathbf{r}_{m,n})\} \\ +C\sum_n\sum_m S_{m,n}\cdot\mathbf{E}_3\cdot\{\exp(i(\mathbf{K}+\mathbf{R}_3)\cdot\mathbf{r}^0_{m,n}) + \exp(i(\mathbf{K}-\mathbf{R}_3)\cdot\mathbf{r}^0_{m,n})\}\end{aligned} \tag{16}$$

Here, this double sum evaluates to zero because $\mathbf{K} \pm \mathbf{R}_j$ $(j = 1{,}3)$ are non-zero vectors and the phase term $[\exp\,(i(\mathbf{K} \pm \mathbf{R}_j) \cdot \mathbf{r}_{m,n})]$ gives destructive interference when (m, n) varies. Therefore,

$$\mathbf{E}_{\text{far}}(\mathbf{K}) = 0 \tag{17}$$

In summary, none of the resonant band edges at the M_1 point radiate into the far field from an unmodulated photonic crystal.

Supplementary Note 3.4. Far-field electric field properties of position-modulated photonic crystals

Next, we introduce position modulation, which enables the light to be radiated in arbitrary directions. With position modulation, the geometric center of each air hole in a photonic crystal changes as follows:

$$\bar{\mathbf{r}}_{m,n} = \mathbf{r}^0_{m,n} + \Delta\mathbf{d}\sin(\mathbf{k}\cdot\mathbf{r}^0_{m,n}) \tag{18}$$

Here, $\Delta\mathbf{d}$ is a vector representing the amplitude and the direction of the shift in position of each air hole, and $\mathbf{k}$ is the diffraction vector. We fix the size of all air holes to $S_{m,n} = S_0$.

(i) Band edges A and B :

By substituting Supplementary Eqs. (5) and (18) into Supplementary Eq. (14), we obtain

$$\mathbf{E}_{\mathrm{far}}(\mathbf{K}) = C\sum_n\sum_m S_0 \cdot \mathcal{E}\left(\mathbf{r}^0_{m,n} + \Delta\mathbf{d}\sin\left(\mathbf{k}\cdot\mathbf{r}^0_{m,n}\right)\right)\exp\left(i\mathbf{K}\cdot\left(\mathbf{r}^0_{m,n} + \Delta\mathbf{d}\sin\left(\mathbf{k}\cdot\mathbf{r}^0_{m,n}\right)\right)\right)$$

$$= 2iCS_0\mathbf{E}_1\sum_n\sum_m (-1)^{m+n}\sin\left(\mathbf{R}_1\cdot\Delta\mathbf{d}\sin\left(\mathbf{k}\cdot\mathbf{r}^0_{m,n}\right)\right)\exp\left(i\mathbf{K}\cdot\mathbf{r}^0_{m,n}\right)\exp\left(i\mathbf{K}\cdot\Delta\mathbf{d}\sin\left(\mathbf{k}\cdot\mathbf{r}^0_{m,n}\right)\right)$$
$$+2iCS_0\mathbf{E}_3\sum_n\sum_m (-1)^{m+n}\sin\left(\mathbf{R}_3\cdot\Delta\mathbf{d}\sin\left(\mathbf{k}\cdot\mathbf{r}^0_{m,n}\right)\right)\exp\left(i\mathbf{K}\cdot\mathbf{r}^0_{m,n}\right)\exp\left(i\mathbf{K}\cdot\Delta\mathbf{d}\sin\left(\mathbf{k}\cdot\mathbf{r}^0_{m,n}\right)\right) \quad (19)$$

Here, we assume that $\Delta\mathbf{d}$ is small, such that $\sin x \sim x$, $\exp(x) \sim 1 + x$, and second powers of $\Delta\mathbf{d}$ are negligible. Using these approximations, we find that

$$\mathbf{E}_{\mathrm{far}}(\mathbf{K}) = 2iCS_0\mathbf{E}_1\sum_n\sum_m (-1)^{m+n}\,\mathbf{R}_1\cdot\Delta\mathbf{d}\sin\left(\mathbf{k}\cdot\mathbf{r}^0_{m,n}\right)\exp\left(i\mathbf{K}\cdot\mathbf{r}^0_{m,n}\right)\left(1 + i\mathbf{K}\cdot\Delta\mathbf{d}\sin\left(\mathbf{k}\cdot\mathbf{r}^0_{m,n}\right)\right)$$
$$+2iCS_0\mathbf{E}_3\sum_n\sum_m (-1)^{m+n}\,\mathbf{R}_3\cdot\Delta\mathbf{d}\sin\left(\mathbf{k}\cdot\mathbf{r}^0_{m,n}\right)\exp\left(i\mathbf{K}\cdot\mathbf{r}^0_{m,n}\right)\left(1 + i\mathbf{K}\cdot\Delta\boldsymbol{d}\sin\left(\boldsymbol{k}\cdot\boldsymbol{r}^0_{m,n}\right)\right) \quad (20)$$
$$= 2iCS_0[(\mathbf{R}_1\cdot\Delta\mathbf{d})\mathbf{E}_1 + (\mathbf{R}_3\cdot\Delta\mathbf{d})\mathbf{E}_3]\sum_n\sum_m (-1)^{m+n}\sin\left(\mathbf{k}\cdot\mathbf{r}^0_{m,n}\right)\exp\left(i\mathbf{K}\cdot\mathbf{r}^0_{m,n}\right)$$

By expressing the sinusoids as complex exponentials and using the relation $(-1)^{m+n} = \exp(-i\mathbf{R}_j\cdot\mathbf{r}^0_{m,n})$ $(j = 1,2,3,4)$, we obtain

$$\mathbf{E}_{\mathrm{far}}(\mathbf{K}) = CS_0[(\mathbf{R}_1\cdot\Delta\mathbf{d})\mathbf{E}_1 + (\mathbf{R}_3\cdot\Delta\mathbf{d})\mathbf{E}_3] \times \sum_n\sum_m\left[\exp\left(i\left(\mathbf{K} - \mathbf{R}_j + \mathbf{k}\right)\cdot\mathbf{r}^0_{m,n}\right) - \exp\left(i\left(\mathbf{K} - \mathbf{R}_j - \mathbf{k}\right)\cdot\mathbf{r}^0_{m,n}\right)\right] \quad (21)$$

Supplementary Eq. (21) expresses total radiation into the far field by the change of the strength of the

electric field radiated at each air hole. Next, we evaluate the double sum when $\mathbf{K} = \mathbf{R}_j \pm \mathbf{k}$, for which the summation terms are finite. Defining the number of photonic crystal period as *N*, we write the result of the double sum as follows:

$$\mathbf{E}_{\mathrm{far}}\left(\mathbf{K} = \mathbf{R}_j \pm \mathbf{k}\right) = C \cdot N^2 \cdot S_0[(\mathbf{R}_1 \cdot \Delta\mathbf{d})\mathbf{E}_1 + (\mathbf{R}_3 \cdot \Delta\mathbf{d})\mathbf{E}_3] \tag{22}$$

If we take the absolute value of $\mathbf{E}_{\mathrm{far}}$, then, since $\mathbf{R}_1$ and $\mathbf{R}_3$ are orthogonal, we get

$$\left|\mathbf{E}_{\mathrm{far}}\left(\mathbf{K} = \mathbf{R}_j \pm \mathbf{k}\right)\right| = C'\,S_0 \cdot |\mathbf{E}_1| \cdot |\mathbf{R}_1| \cdot |\Delta\mathbf{d}| \tag{23}$$

where $C' = N^2 C$. Thus, the amplitude of the electric field in the far field is proportional to the amplitude of position modulation $|\Delta\mathbf{d}|$. If we define $C'' \equiv C' S_0 |\mathbf{E}_1|$ and let $|\Delta\mathbf{d}| = 0.15a$ (as we have used in our position modulated devices), then the far-field energy $P \propto |\mathbf{E}_{\mathrm{far}}|^2 = 0.44 C''^2$.

Additionally, we can obtain radiation in an arbitrary emission direction in free space (polar angle: θ, azimuthal angle: ϕ) by setting the modulation wave vector to

$$\begin{aligned} \mathbf{k} &= \pm\left(\mathbf{K} - \mathbf{R}_j\right) \quad (j = 1,2,3,4) \\ &= \pm\left\{\frac{2\pi}{a} \cdot \frac{1}{\sqrt{2}n_{\mathrm{eff}}}(\sin\theta\cos\phi, \sin\theta\sin\phi) - \frac{2\pi}{a}\left(\pm\frac{1}{2}, \pm\frac{1}{2}\right)\right\} \\ &= \pm\frac{2\pi}{a}\left(\frac{\sin\theta\cos\phi}{\sqrt{2}n_{\mathrm{eff}}} \pm \frac{1}{2}, \ \frac{\sin\theta\sin\phi}{\sqrt{2}n_{\mathrm{eff}}} \pm \frac{1}{2}\right) \end{aligned} \tag{24}$$

This equation is consistent with that which we have reported in our previous work [Supplementary Ref. 4].

(ii) Band edges C and D :

Substituting Supplementary Eqs. (7) and (18) into Supplementary Eq. (14), we obtain

$$\begin{aligned} \mathbf{E}_{\mathrm{far}}(\mathbf{K}) &= C\sum_n\sum_m S_0 \\ &\quad \cdot \mathcal{E}\left(\mathbf{r}_{m,n} + \Delta\mathbf{d}\sin\left(\mathbf{k}\cdot\mathbf{r}^0_{m,n}\right)\right)\exp\left(i\mathbf{K}\cdot\left(\mathbf{r}_{m,n} + \Delta\mathbf{d}\sin\left(\mathbf{k}\cdot\mathbf{r}^0_{m,n}\right)\right)\right) \\ &= 2CS_0\mathbf{E}_1\sum_n\sum_m (-1)^{m+n}\cos\left(\mathbf{R}_1\cdot\Delta\mathbf{d}\sin\left(\mathbf{k}\cdot\mathbf{r}^0_{m,n}\right)\right)\exp\left(i\mathbf{K}\cdot\mathbf{r}^0_{m,n}\right)\exp\,(i\mathbf{K} \\ &\quad \cdot \Delta\mathbf{d}\sin\left(\mathbf{k}\cdot\mathbf{r}^0_{m,n}\right)) \end{aligned} \tag{25}$$

$$+2CS_0\mathbf{E}_3\sum_n\sum_m(-1)^{m+n}\cos\left(\mathbf{R}_3\cdot\Delta\mathbf{d}\sin\left(\mathbf{k}\cdot\mathbf{r}^0_{m,n}\right)\right)\exp\left(i\mathbf{K}\cdot\mathbf{r}^0_{m,n}\right)\exp\left(i\mathbf{K}\cdot\Delta\mathbf{d}\sin\left(\mathbf{k}\cdot\mathbf{r}^0_{m,n}\right)\right)$$

$$\begin{aligned}\mathbf{E}_{\mathrm{far}}(\mathbf{K}) &= 2\mathbf{E}_1CS_0\sum_n\sum_m(-1)^{m+n}\left[1-\frac{\left(\mathbf{R}_1\cdot\Delta\mathbf{d}\sin\left(\mathbf{k}\cdot\mathbf{r}^0_{m,n}\right)\right)^2}{2}\right]\\ &\times\exp\left(i\mathbf{K}\cdot\mathbf{r}^0_{m,n}\right)\times\left(1+i\mathbf{K}\cdot\Delta\mathbf{d}\sin\left(\mathbf{k}\cdot\mathbf{r}^0_{m,n}\right)\right)\\ &+2\mathbf{E}_3CS_0\sum_n\sum_m(-1)^{m+n}\left[1-\frac{\left(\mathbf{R}_3\cdot\Delta\mathbf{d}\sin\left(\mathbf{k}\cdot\mathbf{r}^0_{m,n}\right)\right)^2}{2}\right]\\ &\times\exp\left(i\mathbf{K}\cdot\mathbf{r}_{m,n}\right)\times\left(1+i\mathbf{K}\cdot\Delta\mathbf{d}\sin\left(\mathbf{k}\cdot\mathbf{r}^0_{m,n}\right)\right)\\ &=2Ci(\mathbf{K}\cdot\Delta\mathbf{d})(\mathbf{E}_1+\mathbf{E}_3)S_0\sum_n\sum_m(-1)^{m+n}\sin\left(\mathbf{k}\cdot\mathbf{r}^0_{m,n}\right)\exp\left(i\mathbf{K}\cdot\mathbf{r}^0_{m,n}\right)\end{aligned}\tag{26}$$

As before, we assume that $\Delta\mathbf{d}$ is small. By expressing the sinusoids as complex exponentials and using the relation $(-1)^{m+n}=\exp(-i\mathbf{R}_j\cdot\mathbf{r}^0_{m,n})$ $(j=1,2,3,4)$, we obtain

$$\begin{aligned}\mathbf{E}_{\mathrm{far}}(\mathbf{K}) = {} & CS_0(\mathbf{K}\cdot\Delta\mathbf{d})(\mathbf{E}_1+\mathbf{E}_3)\times\\ & \sum_n\sum_m\left[\exp\left(i\left(\mathbf{K}-\mathbf{R}_j+\mathbf{k}\right)\cdot\mathbf{r}^0_{m,n}\right)-\exp\left(i\left(\mathbf{K}-\mathbf{R}_j-\mathbf{k}\right)\cdot\mathbf{r}^0_{m,n}\right)\right]\end{aligned}\tag{27}$$

Evaluating the double sum when $\mathbf{K}=\mathbf{R}_j\pm\mathbf{k}$ and taking the absolute value of the result yields

$$\left|\mathbf{E}_{\mathrm{far}}\left(\mathbf{K}=\mathbf{R}_j\pm\mathbf{k}\right)\right|=C'\sqrt{2}S_0\cdot|\mathbf{E}_1|\cdot|\mathbf{K}\cdot\Delta\mathbf{d}|\tag{28}$$

In Supplementary Eq. (28), the effect of the change of the strength of radiative electric field at each air hole vanishes, and only the effect of the change of the position of air holes remains. The strength of radiation depends on the radiation direction, and the strength of radiation decreases when the polar angle θ approaches 0° (i.e., when $\mathbf{K}$ approaches zero). For the case in which $\Delta\mathbf{d}$ and $\mathbf{K}$ are orthogonal, $\mathbf{E}_{\mathrm{far}}$ is also zero. For the case in which $\Delta\mathbf{d}$ and $\mathbf{K}$ are parallel, $P\propto|\mathbf{E}_{\mathrm{far}}|^2=0.078C''^2$ (less than 1/5 of that of the band edges A and B) at $\theta=90^{\circ}$, and only $0.0023C''^2$ (less than 1/200 of that of the band edges A and B) at $\theta=10^{\circ}$. Altogether, the strength of the far-field radiation for band edges C and

D is inevitably low.

Supplementary Note 3.5. Far-field electric field properties of size-modulated photonic crystals

Next, we separately introduce size modulation, in which the size of each air hole is changed as follows:

$$\bar{S}_{m,n} = S_0 + \Delta S \sin\left(\mathbf{k} \cdot \mathbf{r}^0_{m,n}\right) \tag{29}$$

By substituting Supplementary Eq. (29) into Supplementary Eq. (14), we obtain

$$\mathbf{E}_{\text{far}}(\mathbf{K}) = C \sum_n \sum_m \left[S_0 + \Delta S \sin\left(\mathbf{k} \cdot \mathbf{r}_{m,n}\right)\right] \boldsymbol{\mathcal{E}}\left(\mathbf{r}^0_{m,n}\right) \exp\left(i\mathbf{K} \cdot \mathbf{r}^0_{m,n}\right) \tag{30}$$

(i) Band edges A and B :

As discussed above, since an electric-field node overlaps each air hole, $\boldsymbol{\mathcal{E}}\left(\mathbf{r}^0_{m,n}\right) = \mathbf{0}$, and no radiation occurs:

$$\left|\mathbf{E}_{\text{far}}\left(\mathbf{K} = \mathbf{R}_j \pm \mathbf{k}\right)\right| = 0 \tag{31}$$

(ii) Band edges C and D :

By substituting Supplementary Eq. (7) into Supplementary Eq. (14),

$$\begin{aligned} \mathbf{E}_{\text{far}}(\mathbf{K}) &= C \sum_n \sum_m \left[S_0 + \Delta S \sin\left(\mathbf{k} \cdot \mathbf{r}^0_{m,n}\right)\right] \boldsymbol{\mathcal{E}}\left(\mathbf{r}^0_{m,n}\right) \exp\left(i\mathbf{K} \cdot \mathbf{r}^0_{m,n}\right) \\ &= 2C\mathbf{E}_1 \sum_n \sum_m (-1)^{m+n} \left[S_0 + \Delta S \sin\left(\mathbf{k} \cdot \mathbf{r}^0_{m,n}\right)\right] \exp\left(i\mathbf{K} \cdot \mathbf{r}^0_{m,n}\right) \\ &\quad + 2C\mathbf{E}_3 \sum_n \sum_m (-1)^{m+n} \left[S_0 + \Delta S \sin\left(\mathbf{k} \cdot \mathbf{r}^0_{m,n}\right)\right] \exp\left(i\mathbf{K} \cdot \mathbf{r}^0_{m,n}\right) \end{aligned} \tag{32}$$

By expressing the sinusoids as complex exponentials and using the relation $(-1)^{m+n} = \exp(-i\mathbf{R}_j \cdot \mathbf{r}_{m,n}) \quad (j = 1,2,3,4)$, we obtain

$$\mathbf{E}_{\text{far}}(\mathbf{K}) = 2C(\mathbf{E}_1 + \mathbf{E}_3) \tag{33}$$

$$\sum_n \sum_m \Bigg[S_0 \exp\left(i(\mathbf{K}-\mathbf{R}_j)\cdot \mathbf{r}^0_{m,n}\right) + \frac{\Delta S}{2i}\exp\left(i(\mathbf{K}-\mathbf{R}_j+\mathbf{k})\cdot \mathbf{r}_{m,n}\right) - \frac{\Delta S}{2i}\exp\left(i(\mathbf{K}-\mathbf{R}_j-\mathbf{k})\cdot \mathbf{r}^0_{m,n}\right)\Bigg]$$

As with Supplementary Eq. (23), we evaluate the double sum, whereupon we obtain

$$\left|\mathbf{E}_{\text{far}}\left(\mathbf{K}=\mathbf{R}_j \pm \mathbf{k}\right)\right| = C'\sqrt{2}\cdot|\mathbf{E}_1|\,|\Delta S| \tag{34}$$

From this equation, we can follow the same steps that led to Supplementary Eq. (24) in order to obtain an electric field in the far field, radiated in an arbitrary direction at a strength proportional to ΔS.

Supplementary Note 3.6. Far-field electric field properties of dually modulated photonic crystals

As discussed in Supplementary Note 3.4 and Supplementary Note 3.5, from photonic crystals with only position modulation or only size modulation, two modes among the four modes A, B, C and D cannot emit to free space, resulting in low radiation efficiency. In order to improve the radiation efficiency, as well as suppress destructive interference to improve the beam quality, we now introduce dual modulation, where the position and size of each air hole are modulated simultaneously.

(i) Band edges A and B :

The far-field electric field is identical to that when only position modulation is considered, i.e.,

$$\left|\mathbf{E}_{\text{far}}\left(\mathbf{K}=\mathbf{R}_j \pm \mathbf{k}\right)\right| = C'\,S_0\cdot|\mathbf{E}_1|\cdot|\mathbf{R}_1|\cdot|\Delta\mathbf{d}| \tag{35}$$

Thus, light is radiated in an arbitrary direction at a strength proportional to the position modulation amplitude $|\Delta\mathbf{d}|$. Setting $|\Delta\mathbf{d}| = 0.08a$ (the value used for our dually modulated photonic crystals in the main text), we estimate that the radiative power $P \propto |\mathbf{E}_{\text{far}}|^2 = 0.13C''^2$.

(ii) Band edges C and D :

By substituting Supplementary Eqs. (18) and (24) into Supplementary Eq. (14),

$$\mathbf{E}_{\mathrm{far}}(\mathbf{K}) = C\sum_{n}\sum_{m}\left[S_0 + \Delta S\sin\left(\mathbf{k}\cdot\mathbf{r}^0_{m,n}\right)\right]\boldsymbol{\mathcal{E}}\left(\mathbf{r}^0_{m,n} + \Delta\mathbf{d}\sin\left(\mathbf{k}\cdot\mathbf{r}^0_{m,n}\right)\right)$$

$$\times \exp\left(i\mathbf{K}\cdot\left(\mathbf{r}^0_{m,n} + \Delta\mathbf{d}\sin\left(\mathbf{k}\cdot\mathbf{r}^0_{m,n}\right)\right)\right)$$

$$= CS_0\sum_{n}\sum_{m}\boldsymbol{\mathcal{E}}\left(\mathbf{r}_{m,n} + \Delta\mathbf{d}\sin\left(\mathbf{k}\cdot\mathbf{r}^0_{m,n}\right)\right)\exp\left(i\mathbf{K}\cdot\left(\mathbf{r}^0_{m,n} + \Delta\mathbf{d}\sin\left(\mathbf{k}\cdot\mathbf{r}^0_{m,n}\right)\right)\right) \tag{36}$$

$$+ \Delta S\sum_{n}\sum_{m}\sin\left(\mathbf{k}\cdot\mathbf{r}^0_{m,n}\right)\boldsymbol{\mathcal{E}}\left(\mathbf{r}^0_{m,n} + \Delta\mathbf{d}\sin\left(\mathbf{k}\cdot\mathbf{r}^0_{m,n}\right)\right)$$

$$\times \exp\left(i\mathbf{K}\cdot\left(\mathbf{r}^0_{m,n} + \Delta\mathbf{d}\sin\left(\mathbf{k}\cdot\mathbf{r}^0_{m,n}\right)\right)\right)$$

The first double sum on the right-hand side represents the net effect of position modulation, which is very small for modes C and D, as previously discussed in Supplementary Note 3.4, and thus it can be ignored. The second double sum represents size modulation. By ignoring terms with the product $\Delta S\Delta\mathbf{d}$ in this sum, the sum becomes

$$\left|\mathbf{E}_{\mathrm{far}}\left(\mathbf{K} = \mathbf{R}_j \pm \mathbf{k}\right)\right| = C'\sqrt{2}\cdot|\mathbf{E}_1|\,|\Delta S| \tag{37}$$

which is identical to the amplitude of the far-field electric field when only size modulation is used. Thus, light is radiated in an arbitrary direction at a strength proportional to the amplitude of size modulation ΔS. Setting $\Delta S = 0.03a^2$ (corresponding to our dually modulated devices in the main text), we can estimate that the radiated power $P \propto |\boldsymbol{E}_{\mathrm{far}}|^2 = 0.14C''^2$, which is in quantitative agreement with the results of our calculations by coupled-wave theory in the main text.

In this way, by using the nano-antenna theory formulated in this work, we have shown that our concept of dual modulation, in contrast to either of its constituent, single-modulation methods, enables finite radiation of all four band-edge modes to free space, which is important for high-power operation with high beam quality.

Supplementary Note 4. Parameters for calculations based on coupled-wave theory and the estimation of slope efficiency

The laser structure to be studied is listed in Supplementary Table 1. In the calculation based on coupled-wave theory, we utilized a superlattice with an adjustable periodicity to accommodate the additional coupling introduced by the modulation, including that involving the diffraction vector **k**. In the calculation of Fig. 3 in the main text, we considered an infinite structure with radiation at a polar angle of $\theta \sim 36^{\text{o}}$ and an azimuthal angle of $\phi = 0^{\text{o}}/180^{\text{o}}$ in free space. In this example, the modulated PCSEL can be treated as a superlattice with a periodicity of $8a \times 2a$. Similar characteristics are also obtained for other radiation angles, which can be calculated by using superlattices with different periodicities.

For the estimation of the slope efficiency, we considered a finite-size device with a circular electrode diameter of 100 μm, and we calculated a lasing mode radiation constant α_v (=18.3cm^{-1}) and in-plane loss $\alpha_{//}$ (=7.6cm^{-1}). Then, we calculated the slope efficiency for upward emission with the following equation:

$$\eta_{up} = \frac{\hbar\omega}{e}\frac{\frac{1}{2}\alpha_v}{\alpha_v + \alpha_{//} + \alpha_0}. \tag{38}$$

Assuming a material loss of α_0 =5 cm^{-1}, we obtained a slope efficiency for upward emission of 0.39 W/A.

Supplementary Table 1. Structural parameters of the dually modulated photonic crystal lasers.

Layer	Thickness (nm)	Refractive index
n-clad (AlGaAs)	2000	3.12
AlGaAs	80	3.45
Active (InGaAs/AlGaAs)	90	3.51
AlGaAs	25	3.45
GaAs	65	3.55
Photonic crystal	150	3.39
p-clad (AlGaAs)	2000	3.30

Supplementary Note 5. Fabrication process of on-chip dually modulated photonic crystal lasers array

We describe the fabrication process of an on-chip dually modulated photonic crystal laser array. A schematic diagram of the fabrication process is shown in Supplementary Fig. 8. First, 5μm of n-GaAs with a carrier concentration of $\sim 1\times 10^{18}$cm^{-3} was grown on a semi-insulating (SI) GaAs substrate by MOVPE. The n-GaAs was used for lateral current injection. Then, a 1μm-thick n-AlGaAs cladding layer and InGaAs/AlGaAs MQW were grown. Afterward, approximately 250nm of p-GaAs used to fabricate the photonic crystal air-holes was grown atop the MQW. Dually modulated photonic crystals were patterned into the p-GaAs layer by a combination of electron-beam lithography and reactive ion etching methods. The lattice constant of the photonic crystals were set to approximately 195nm, where the wavelength of the M-point matches the emission wavelength of the MQW. Then, 1μm-thick p-AlGaAs cladding layer was regrown over the photonic crystal air holes. Under appropriate regrowth conditions, the air holes were successfully embedded into the p-GaAs layer [Supplementary Ref. 5]. After embedding the air-holes with the p-cladding layer, a p^+-GaAs capping layer was grown. A schematic of the laser wafer is shown in Supplementary Fig. 8(a).

Next, a mesa array was fabricated by a combination of photolithography and dry etching with a Cl_2/BCl_3 gas mixture. Then, isolation grooves were etched down to the SI-GaAs substrate in the same manner as the mesa etching process. SiN_x was next deposited atop the structure as an insulating layer using SiH_4 plasma by PE-CVD (Supplementary Fig. 8(b)). Then, vias for the n- and p-contact electrodes were etched into the SiN_x by a combination of photolithography and fluorine-based dry etching. Afterward, Ti/Au p-contact and Ge-Au/Ni/Au n-contact electrodes were deposited over the vias (Supplementary Fig. 8(c)), followed by Ti/Au n- and p-line electrodes. We note that we re-deposited SiN_x in between the n- and p- line electrodes for the purpose of electrical insulation (Supplementary Fig. 8(d)). Finally, an additional layer of SiN_x was deposited on the surface of the SI-GaAs substrate to serve as an anti-reflection layer, preventing the unwanted reflection of light exiting the device.

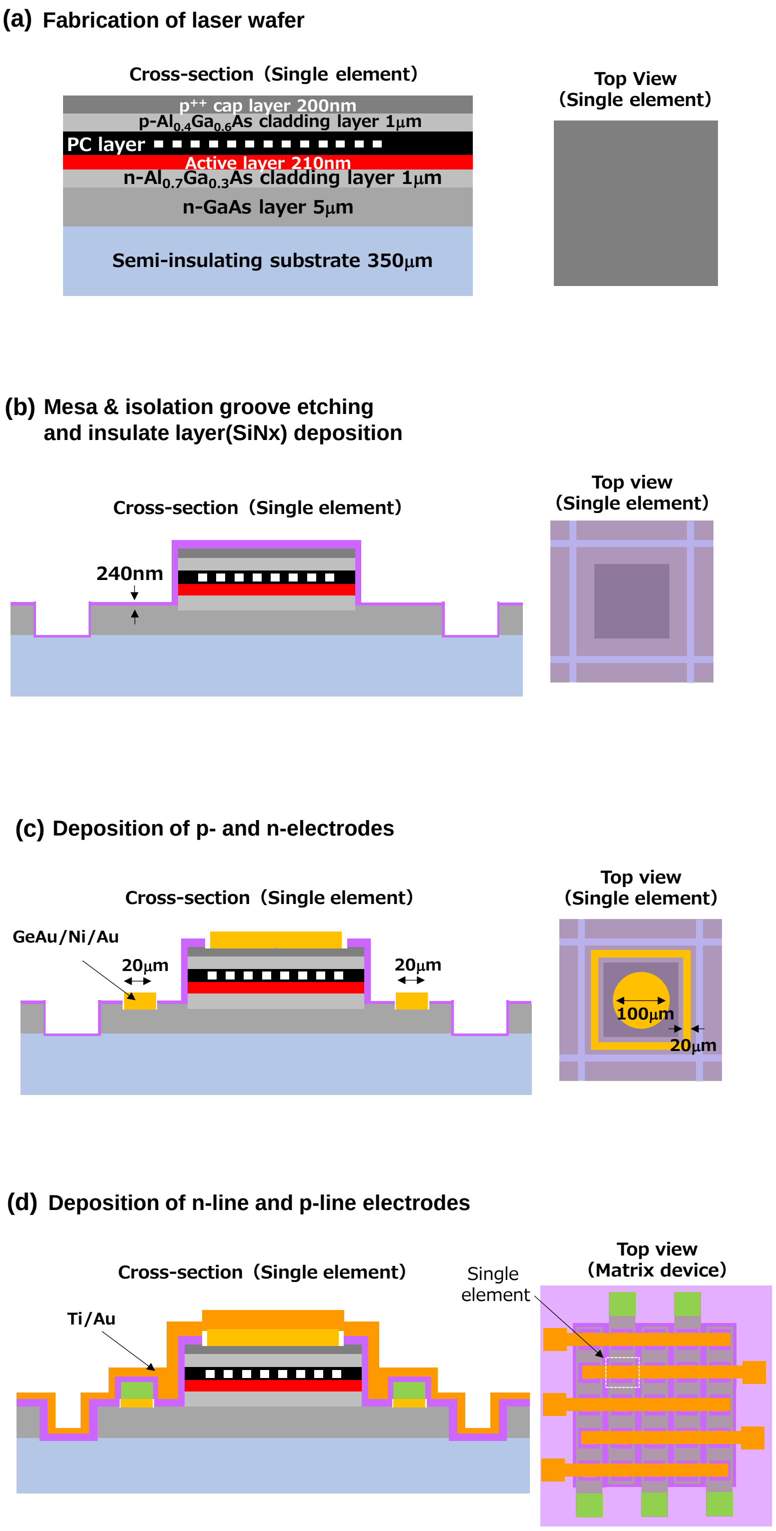

Supplementary Figure 8. Schematic diagram of the fabrication process of an on-chip dually modulated photonic crystal laser array.

Supplementary Note 6. Operation details of real time 2D beam scanning

We describe the real time 2D beam of on-chip dually modulated photonic crystal laser array. For switching the laser elements, we used micro-controllers programmed by a computer. We have performed operations include single scanning of polar angle (or azimuthal angle) with fixed azimuthal angle (or polar angle), parallel scanning of polar angle (or azimuthal angle) with two different fixed azimuthal angles (or polar angles), and also the combinations thereof. Operation details are given in Supplementary Table 2 below. The 2D beam scanning is provided as a Supplementary Movie 1.

Supplementary Table 2. Operation details of 2D beam scanning

Elapsed time (s)		θ (°)	ϕ (°)	step angle (°)	
0 - 4		0 to 45	90/270 (fix)	5	single
4 - 8		0~45 &	90/270 (fix)	5	parallel
		11~20	0/180 (fix)	1	
8 - 12		0~45 &	90/270 (fix)	5	parallel
		20 (fix)	0 to 45	1	
12 - 16		0~45	90/270 (fix)	5	parallel
		0~45	0/180 (fix)	5	
16 - 19		0~45	0/180 (fix)	5	single
19 - 21.5		0~45	90/270 (fix)	5	single
21.5 - 24.5		0~45	0/180 (fix)	5	parallel
		11~20	90/270 (fix)	5	
24.5 - 27.5		0~45	90/270 (fix)	5	parallel
		11~20	0/180 (fix)	5	
27.5 - 32.5	(i)	0~20	90/270 (fix)	5	single
	(ii)	25~45	90/270 (fix)	5	parallel
		20 (fix)	65/245~45/225	5	
	(iii)	40~0	90/270 (fix)	5	parallel
		20 (fix)	40/220~0/180	5	
32.5 - 38	(i)	0~20	0/180 (fix)	5	single
	(ii)	25~45	0/180 (fix)	5	parallel
		20 (fix)	25/205~45/225	5	
	(iii)	40~0	0/180 (fix)	5	parallel
		20 (fix)	45/225~90/270	5	

Elapsed time (s)		θ (°)	ϕ (°)	step angle (°)	
38 - 40.5	(i)	0~20	0/180 (fix)	5	single
	(ii)	25~45	0/180 (fix)	5	parallel
		16~20	0/180 (fix)	1	
40.5 - 43.5	(i)	0~20	90/270 (fix)	5	single
	(ii)	25~45	90/270 (fix)	5	parallel
		16~20	90/270 (fix)	1	
43.5 - 46.5		0~45	0/180 (fix)	5	parallel
		11~20	90/270 (fix)	1	
46.5 - 49		0~45	90/270 (fix)	5	parallel
		11~20	0/180 (fix)	1	
49 - 54.5	(i)	0~20	0/180 (fix)	5	parallel
		0~20	90/270 (fix)	5	
	(ii)	25~45	0/180 (fix)	5	parallel
		20 (fix)	65/245~45/225	5	
	(iii)	40~0	0/180 (fix)	5	parallel
		20 (fix)	40/220~0/180	5	
54.5 - 59.5	(i)	0~20	0/180 (fix)	5	parallel
		0~20	90/270 (fix)	5	
	(ii)	25~45	90/270 (fix) &	5	parallel
		20 (fix)	25/205 to 45/225	5	
	(iii)	40~0	90/270 (fix)	5	parallel
		20 (fix)	45/225~90/270	5	

Supplementary Note 7. Device structure for beam scanning with a much larger number of resolvable points

In this section, we describe a device structure for increasing the number of resolvable points without requiring a proportionally large increase of the total device size. As an example, the number of resolvable points achievable with this device can be increased from 100 to 90,000 (by a factor of 900) while limiting the increase of the device size to merely a factor of 4. Furthermore, each excitation area can be maintained at 100μm×100μm, so a narrow divergence angle and a high, watt-class (or even higher) output power can be preserved. The details are described below.

Supplementary Fig. 9 show the schematic device structure. In this device, a N×N array of small electrodes is formed on the bottom of a device which possesses a dually modulated photonic crystal whose **k** vectors are gradually varied in the *x-y* plane. Isolation mesas are not formed in order to allow current to spread from the area of each electrode into those of its adjacent electrodes. In this structure, the current injected by adjacent electrodes driven in tandem is shared over a single, unified area causing the device to emit single far-field. The individual electrodes are assumed to be controlled by a matching N×N transistor matrix array. By exciting multiple electrodes (for example, M×M electrodes among N×N: $\mathrm{E}_{i,j}$ to $\mathrm{E}_{i+\mathrm{M},\ j+\mathrm{M}-1}$) simultaneously, and by exciting sets of M×M electrodes incrementally along *x* and *y*, it is possible to steer the beam very finely. In this way, the number of independent, resolvable points can be increased significantly without needing to increase the total device size very much.

A more concrete explanation is as follows. We assume that the total device size is 6mm x 6mm, in which diffraction vector **k** is changed gradually to enable beam scanning over a total range of ±30° field of view. We set the area of each electrode to 10μm×10μm and arrange these electrodes with a period of 20μm in the *x*- and *y*-directions. We inject current into a set of 5×5 electrodes as a single unit, which excites a 100μm×100μm area in order to produce one far-field beam. Then, we move onto the next set of 5×5 electrodes, redirecting current from a row of 5 electrodes on one side to the row of 5 electrodes on the opposite side, thereby shifting the entire set by 20μm along *x* and/or *y*. In this case, the number of resolvable points increases to 300 and the angle separation narrows to 0.1° along each of *x* and *y*. Therefore, in total, 300×300=90,000 resolvable points can be obtained from a device area of 6mm×6mm;

that is, the number of resolvable points can be increased from 100 to 90,000 (a factor of 900) while the increase in device size is limited to a factor of 4. Furthermore, because each excitation area is maintained at 100μm×100μm, a narrow divergence angle and a high, watt-class (or even higher) output power can be preserved.

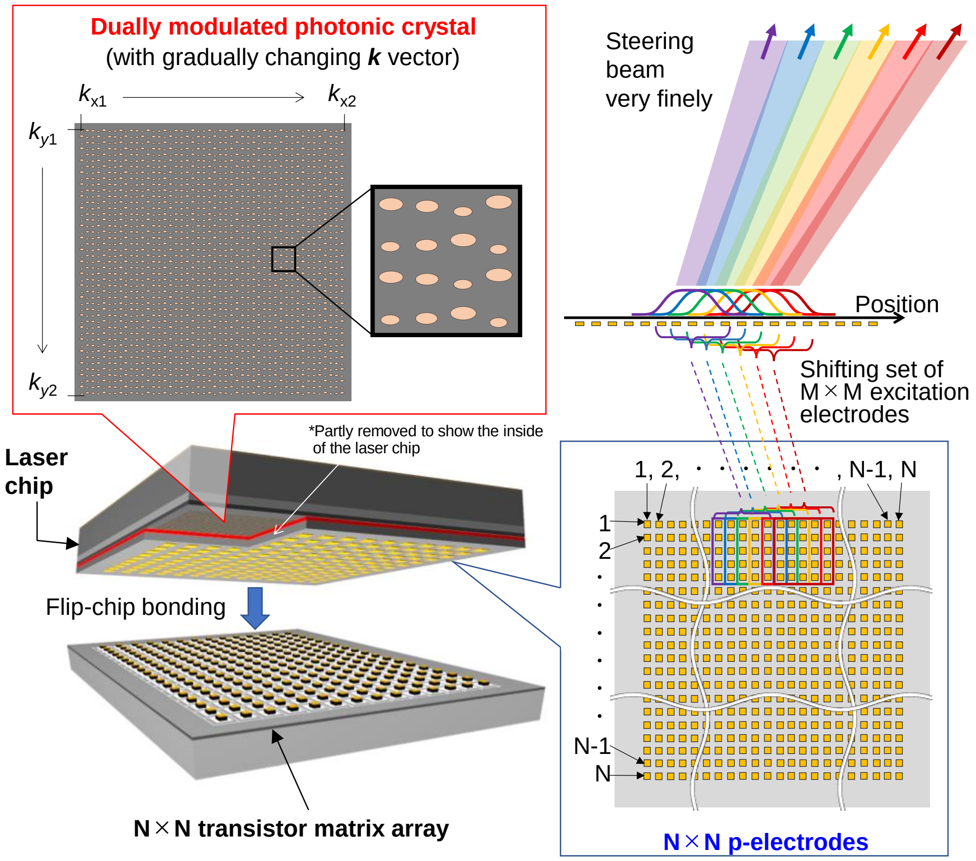

Supplementary Figure 9. Schematic device structure for beam scanning operation with a much larger number of resolvable points, but without a proportionally large increase of the total device size. By exciting multiple electrodes (for example, a set of M×M electrodes among N×N) simultaneously, and by shifting the sets of electrodes incrementally along *x* and *y*, it is possible to steer the beam very finely.

Supplementary Note 8. Concept of a new, combined flash- and scanning-type LiDAR system

In this section, we explain the concept of a unique new LiDAR system, in which the benefits of flash-type and beam-scanning-type ToF LiDARs are integrated, and to which a configuration with 100 resolvable points, as demonstrated in this work, can be applied. The schematic of this unique LiDAR system is shown in Supplementary Fig. 10. A conventional flash-type LiDAR system for ranges of 10~20m suffers from the critical issue of struggling to range low-reflectivity objects, such as a black metallic car. This is because the intensity of the reflected light is very low due to the inherent nature (namely, the low irradiation power density) of the flash-type LiDAR system, which simultaneously irradiates all objects over a wide viewing field, and thus has an insufficient S/N ratio for ranging the low-reflectivity objects (see Supplementary Fig. 10(a)). However, by combining our device with the flash-LiDAR system as shown in Supplementary Fig. 10(b), we can selectively irradiate the low-reflectivity object with sufficient optical power, once it is discovered in a camera image obtained using the flash-type LiDAR system. The ranging of low-reflectivity objects would thus be possible. If multiple low-reflectivity objects are detected, then we can use the parallel beam scanning functionality of our device to detect them simultaneously.

We note that, in the above new application, beams with even wide divergence angles may be permissible, because the ToF camera of flash-type LiDAR system possesses angular-resolvable functionality. For example, assuming that we wish to cover a ±30° field of view with a 10×10 array (= 100 resolvable points), as we do with our device in this work, we may simply shrink the size of each element of this array to 30μm×30μm, which would widen the beam divergence angle to ~3° but also reduce the total device size by a factor of ~9. (If watt-class power is required to be maintained, then sets of 3×3 electrodes, each of 30μm×30μm size, can be operated simultaneously.) We are considering this unique LiDAR system as a new implementation of LiDAR technology.

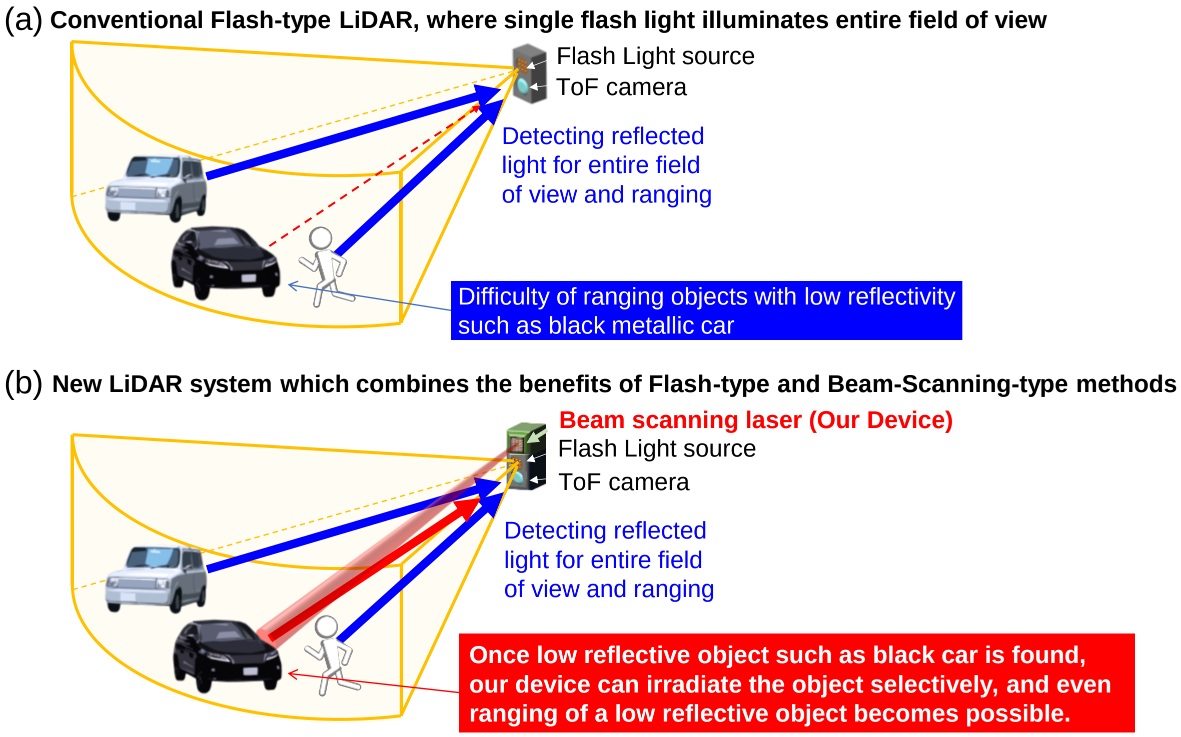

Supplementary Figure 10. (a) Issue of conventional flash-type LiDAR, and (b) merits of combining flash-type LiDAR and beam-scanning-type LiDAR based on our device.

Supplementary Note 9. Estimation of emission angle change with respect to temperature

In this section, we calculate the emission angle change of grating couplers and dually modulated photonic crystal lasers with respect to temperature.

Grating couplers

First, we discuss the emission angle change of grating couplers with respect to temperature. The emission angle θ_g of a grating coupler at a given temperature is given by Supplementary Eq. (39) [Supplementary Ref. 6].

$$\sin\theta_\mathrm{g} = \frac{\Lambda n_\mathrm{eff} - \lambda}{\Lambda} \tag{39}$$

Here, Λ is the grating period, n_eff is the effective refractive index of the grating, and λ is the wavelength. When the temperature of the grating changes, the grating period and the effective refractive index change, as well. On the other hand, for the common case in which an external laser source is used, the wavelength remains constant. Thus, the change of emission angle with respect to the temperature T is given by:

$$\frac{\Delta\theta_\mathrm{g}}{\Delta T} = \frac{1}{\cos\theta_\mathrm{g}}\left(\frac{\Delta n_\mathrm{eff}}{\Delta T} + \frac{\lambda}{\Lambda}\frac{(\Delta\Lambda/\Lambda)}{\Delta T}\right) \tag{40}$$

The grating period changes due to thermal expansion of the semiconductor material used for the gratings. For example, the thermal expansion coefficient of silicon is 2.6×10^{-6} K^{-1}. Thus, $(\Delta\Lambda/\Lambda)/\Delta T$ = 2.6×10^{-6} K^{-1} for silicon gratings. Meanwhile, the thermo-optic coefficient $\Delta n/\Delta T$ of silicon is ~2×10^{-4} K^{-1} [Supplementary Ref. 7]. Assuming the effective refractive index of the grating coupler is ~3, $\Delta n_\mathrm{eff}/\Delta T$ ≈ 1.5×10^{-4} K^{-1}, which is two orders of magnitude larger than $(\Delta\Lambda/\Lambda)/\Delta T$. Thus, the change of emission angle mostly depends on the change of refractive index for silicon gratings.

$$\frac{\Delta\theta_\mathrm{g}}{\Delta T} \approx \frac{1}{\cos\theta_\mathrm{g}}\left(\frac{\Delta n_\mathrm{eff}}{\Delta T}\right) \tag{41}$$

For example, given an emission angle of 10°, $\Delta\theta_\mathrm{g}/\Delta T$ is calculated to be ~9×10^{-3} °K^{-1}.

Dually modulated photonic crystal lasers

Next we discuss the emission angle change of dually modulated photonic crystal lasers with respect to temperature. As mentioned in the manuscript, the vector $\mathbf{K}$ determines the emission direction in free space. In terms of polar emission angle θ and azimuthal emission angle ϕ :

$$\mathbf{K} = \frac{2\pi}{\lambda}(\sin\theta\cos\phi\,,\ \sin\theta\sin\phi)$$
$$|\mathbf{K}| = \frac{2\pi}{\lambda}\sin\theta \tag{42}$$

$\mathbf{K}$ is formed by modulating the photonic crystal lattice points, and its magnitude is inversely proportional to the photonic crystal lattice constant a (i.e., $|\mathbf{K}| \propto 1/a$). As with the silicon grating couplers discussed above, the effect of thermal expansion (i.e., change of a) of the gallium arsenide photonic crystal is two orders of magnitude smaller than its refractive index change. Therefore, $|\mathbf{K}|$ is considered to remain constant with temperature. However, unlike with grating couplers, the change of refractive index with temperature now affects the emission wavelength λ. This implies that the polar emission angle changes by:

$$|\mathbf{K}|\frac{\Delta\lambda}{\Delta T} \approx 2\pi\cos\theta\frac{\Delta\theta}{\Delta T} \tag{43}$$

Substituting $|\mathbf{K}|$ in Supplementary Eq. (42) into Supplementary Eq. (43), we obtain:

$$\frac{\Delta\theta}{\Delta T} \approx \tan\theta\frac{1}{\lambda}\frac{\Delta\lambda}{\Delta T} \tag{44}$$

The change of the emission wavelength of our photonic crystal lasers with respect to temperature ($\Delta\lambda/\Delta T$) is found in experiments to be 0.086 nm K^{-1}. Thus, given the same emission angle of 10° as above, $\Delta\theta/\Delta T$ is calculated to be ~9×10^{-4} ^{o}K^{-1}. This is one order of magnitude smaller than the grating couplers.

Supplementary References

1. Kurosaka, Y., Iwahashi, S., Liang, Y., Sakai, K., Miyai, E., Kunishi, W., Ohnishi, D., and Noda, S. On-chip beam-steering photonic-crystal lasers. *Nat. Photonics* **4**, 447–450 (2010).
2. Yoshida, M., De Zoysa, M., Ishizaki, K., Tanaka, Y., Kawasaki, M., Hatsuda, R., Song, B.-S., Gelleta, J., and Noda. S. Double-lattice photonic-crystal resonators enabling high-brightness semiconductor lasers with symmetric narrow-divergence beams. *Nat. Materials* **18**, 121-128 (2019).
3. Xie, Y.-Y., Ni, P.-N., Wang, Q.-H., Kan, Q., Briere, G., Chen, P.-P., Zhao, Z.-Z., Delga, A., Ren, H.-R., Chen, H.-D., Xu, C., and Genevet, P. Metasurface-integrated vertical cavity surface-emitting lasers for programmable directional lasing emissions. *Nat. Nanotechnol.* **15**, 125-130 (2020).
4. Noda, S., Kitamura, K., Okino, T., Yasuda, D., and Tanaka, Y. Photonic-crystal surface-emitting lasers: Review and introduction of modulated-photonic crystals. *IEEE J. Sel. Top. Quant. Electron.* **23**, 4900107 (2017).
5. Yoshida, M., Kawasaki, M., De Zoysa, M., Ishizaki, K., Hatsuda, R., and Noda, S. Fabrication of photonic crystal structures by tertiary-butyl arsine-based metal–organic vapor-phase epitaxy for photonic crystal lasers. *Appl. Phys. Express* **9**, 062702, (2016).
6. Hulme, J.-C., Doylend, J.-K., Heck M.-J.-R., Peters, J.-D., Davenport, M.-L., Bovington, J.-T., Coldren, L.-A., and Bowers, J.-E., Fully integrated hybrid silicon two dimensional beam scanner *Optics Express*. **23**, 5861-5874 (2015).
7. Komma, J., Schwarz, C., Hofmann, G., Heinert, D., and Nawrodt, R., Thermo-optic coefficient of silicon at 1550 nm and cryogenic temperatures *Appl. Phys. Lett*. **101**, 041905 (2012).
