# Supplementary material for: Dually modulated photonic crystals enabling high-power high-beam-quality two-dimensional beam scanning lasers
Source: Nat Commun. 2020 Jul 17;11:3487. doi: 10.1038/s41467-020-17092-w (PMC7367876; doi:10.1038/s41467-020-17092-w)
Supplement: Supplementary file 3 — Description of Additional Supplementary Files [file 41467_2020_17092_MOESM3_ESM.pdf]

Description of Additional Supplementary Files

File Name: Supplementary Movie 1
Description: **Real-time video of 2D beam scanning** performed in sequence and parallel over not only the range of angles displayed in Fig. 6 of the main text, but also various other angles. Operations include sequential scanning of polar (or azimuthal) angle with fixed azimuthal (or polar) angle, parallel scanning of polar (or azimuthal) angle with two different fixed azimuthal (or polar) angles, and combinations thereof. The operation details are given in Supplementary Table 2 of Supplementary Note 4.
